# Comparative analysis of physicochemical properties, functional activities, and metabolomics of *Annona squamosa* juice fermented by different functional microorganisms

**DOI:** 10.1016/j.fochx.2026.104287

**Published:** 2026-08-22

**Authors:** Yi Deng, Richan Fang, Jun Guo, Chunhui Miao, Yajuan Chen, Xiaohuan Ke, Jianmin Tang, Jiating Lin, Youlin Cheng, Cuixia Zhao, Jian Xiong, Xianyu Deng, Weiwei Li, Conghui Ji, Kai Qian

**Affiliations:** a Department of Thoracic Surgery, The First People's Hospital of Yunnan Province, Kunming University of Science and Technology Affiliated Hospital＆Faculty of Life Science and Technology, Kunming University of Science and Technology, Kunming, 650500, China; b Department of Pulmonary and Critical Care Medicine The First People's Hospital of Yunnan Province Kunming University of Science and Technology Affiliated Hospital, Kunming 650032, China; c Institute of Economic Animal, Chongqing Academy of Animal Sciences, Rongchang, Chongqing 402460, China; d Institute of Sericulture and Apiculture, Yunnan Academy of Agricultural Sciences, Mengzi 661101, China; e Yunnan Zhongfeng Technology Development Co. LTD, Kunming, Yunnan 651701, China; f Department of Modern Agriculture, Zunyi Vocational and Technical College, Zunyi, Guizhou 563000, China; g Yunnan Key Laboratory of Biodiversity Information, Kunming Institute of Zoology, The Chinese Academy of Sciences, Kunming 650201, China

**Keywords:** *Annona squamosa*, Microbial fermentation, Untargeted metabolomics, Enzyme inhibition, Electronic tongue, Functional beverage

## Abstract

*Annona squamosa* is a tropical fruit with unique flavor but rapid postharvest spoilage. This study evaluated fermentation with *Bacillus subtilis*, *Acetobacter tropicalis*, *Limosilactobacillus reuteri*, and *Lactobacillus buchneri* on juice properties. Fermentation increased total phenolics, flavonoids, and free amino acids, and improved taste. Fermented juices showed enhanced antioxidant, antimicrobial, and anti-inflammatory activities, with increased inhibition of α-amylase, α-glucosidase, xanthine oxidase, and pancreatic lipase (*p* < 0.001). Metabolomics revealed differential metabolites linked to organic acid and amino acid metabolism. Epothilone A, anthocyanin derivatives, protocatechuic acid, and γ-glutamylmethionine increased after fermentation. Correlation analysis linked enhanced activities to phenolic compounds and amino acid derivatives. Overall, fermentation improved nutritional, sensory, and functional properties of *A. squamosa* juice, supporting its potential as a functional beverage. Further in vivo studies are needed to confirm these findings.

## Highlights

• Four functional microorganisms were applied to ferment *Annona squamosa* juice.
• Fermentation enhanced phenolic compounds, flavonoids, amino acids, and antioxidant capacity.
• Electronic tongue analysis revealed improved sensory properties with increased sourness, sweetness, and umami.
• Fermented juices exhibited enhanced antimicrobial activity and in vitro enzyme inhibitory properties.
• Untargeted metabolomics revealed strain-dependent metabolic remodeling associated with improved functional properties.

## 1 Introduction

*Annona squamosa L. (A. squamosa)*, commonly known as sugar-apple, belongs to the genus *Annona* in the family *Annonaceae*. It is a tropical fruit tree native to South America and is now widely cultivated in tropical and subtropical regions across Asia, Africa, and the Americas(Moussa et al., 2024). *A. squamosa* is widely appreciated for its distinctive sweet flavor and soft, creamy texture. It is rich in nutrients and bioactive compounds, including vitamins, minerals, dietary fiber, and phytochemicals(Kaur & Kapoor, 2001) such as phenolic compounds, alkaloids, and acetogenins(George et al., 2015). These constituents contribute to its potential health-promoting properties, including antioxidant, anti-inflammatory, antimicrobial, and potential biological activities(Adewole & Ojewole, 2009; Pinto et al., 2005). However, the fruit exhibits a high respiration rate and rapid postharvest ripening, leading to softening and spoilage shortly after harvest, which results in poor storage stability and a short shelf life(Kader, 2002).In addition, its utilization remains largely limited to fresh consumption, with relatively few processed products and underdeveloped deep-processing technologies, thereby restricting industrial development and value addition(Pareek & Yahia, 2011). Therefore, developing novel processing strategies to improve the utilization efficiency and added value of *A. squamosa* is of great importance for the sustainable development of its industry(Fao, 2020).

In recent years, growing consumer demand for healthy foods has driven rapid expansion of the functional fermented beverage market. Compared with traditional dairy-based fermented products, plant-based fermented beverages offer advantages such as being lactose-free, vegan-friendly, and suitable for individuals with specific dietary needs(Marco et al., 2017). Fruits are considered ideal fermentation substrates due to their natural abundance of fermentable sugars, vitamins, minerals, and antioxidants(Dou, Hu, et al., 2025; Dou, Tian, et al., 2025), which support microbial growth and enhance nutritional quality. Among them, *A. squamosa* is particularly suitable for fermentation due to its relatively high sugar content and moderate pH. *Lactic acid* fermentation has been widely recognized as an effective strategy to improve the functional properties of fruit-based products. Previous studies have demonstrated that microbial fermentation can enhance antioxidant capacity, improve sensory properties, and extend shelf life (Filannino et al., 2018). Different microbial strains exhibit distinct metabolic capabilities in fruit fermentation systems. *Bacillus subtilis possesses the ability to produce hydrolytic enzymes that promote substrate degradation*(Stein, 2005); *Acetobacter tropicalis* can oxidize ethanol to produce acetic acid, thereby improving flavor(Gullo & Giudici, 2008)；*Limosilactobacillus reuteri* (Peng et al., 2023) and *Lactobacillus buchneri* (Pérez-Díaz et al., 2023) exhibit probiotic regulatory functions and adaptability to plant-based substrates, respectively. Increasing evidence suggests that different microorganisms can induce distinct metabolic responses in the same fruit matrix, resulting in significant variations in nutritional composition and functional properties of the final products(Luo et al., 2024).

Safety evaluation of microbial strains is a prerequisite in the development of fermented foods. Food-grade microorganisms should be non-pathogenic, non-toxic, and without undesirable metabolite production, such as biogenic amines(Elsaadany et al., 2024). Therefore, preliminary safety evaluation of candidate strains, including hemolytic activity and antibiotic susceptibility assessments, is important before their application in food fermentation(García et al., 2024). Based on this, the present study employed four functional microbial strains to ferment *A. squamosa* juice. The effects of different strains on the physicochemical properties, functional activities, flavor characteristics, and metabolomic profiles of fermented juice were systematically investigated, aiming to elucidate the regulatory mechanisms of microbial fermentation and provide a theoretical basis for the development of functional fermented *A. squamosa* beverages.

## 2 Materials and methods

### 2.1 Test material

#### 2.1.1 Test sample

*A. squamosa* fruits were purchased from a local agricultural market in Kunming, China. Fresh fruits with uniform maturity, intact appearance, and no visible mechanical damage or disease symptoms were selected and immediately processed for fermentation experiments. The microbial strains used in this study were previously isolated from bee-derived products by our research group and are currently preserved in the China Center for Type Culture Collection (CCTCC). The strains included *Bacillus subtilis* (*B. subtilis* preservation number: CCTCC AB 2025223), *Acetobacter tropicalis* (*A. tropicalis* preservation number: CCTCC AB 2026003), *Limosilactobacillus reuteri* (*L. reuteri* preservation number: CCTCC AB 2026004), and *Lactobacillus buchneri* (*L. buchneri* preservation number: CCTCC AB 2025224). Strain selection was based on food fermentation potential, physiological characteristics, enzymatic properties, and preliminary adaptability to *A. squamosa* juice fermentation. The safety of the strains was preliminarily evaluated through hemolytic activity assays and antibiotic susceptibility testing, among other methods(Kim et al., 2024). The four selected strains showed good growth adaptability in *A. squamosa* juice, indicating their potential suitability for fermentation applications.

#### 2.1.2 Main instruments and reagents

DNS reagent(Beijing Solarbio Science & Technology Co., Ltd.)；pNPG Cas:2492-87-7, ovalbumin Cas:9006-59-1, FeCl_2_•4H_2_O Cas:13478–10-9(Shanghai Macklin Biochemical Co., Ltd.)；Ferrozine Cas:69898–45-9(Phygene Biotechnology Co., Ltd.)；citric acid, allopurinol (Tianjin Fengchuan Chemical Reagent Technology Co., Ltd.); starch soluble, sodium sulfite, sodium phosphate (Tianjin Damiao Chemical Reagent Factory); xanthine Cas:69–89-6, α-Amylase Cas:9000-90-2, α-Glucosidase Cas:9001-42-7, orlistat Cas:96829–58-2(Shanghai Yuanye Bio-Technology Co., Ltd.)；acarbose Cas: 56180–94-0 (Rhawn Reagent Co., Ltd., Shanghai, China);xanthine oxidase Cas:9002-17-9, pancreatic lipase Cas:9001-62-1 (Shanghai Shifeng Biological Products Co., Ltd.); Tris-HCl buffer, dimethyl sulfoxide, p-nitrophenol, *p*-nitrophenyl butyrate (Hefei Jinu Biotechnology Co., Ltd.); antibiotics (Bikeman Biotechnology Co., Ltd.); Total Phenols (TP) Assay Kit G0117W, Total Flavonoids Assay Kit G0118W, ABTS Free Radical Scavenging Capacity Assay Kit G0127W, DPPH Free Radical Scavenging Capacity Assay Kit G0128W, Amino Acid (AA) Content Determination Assay Kit G0415W (Suzhou Grees Bio-Technology Co., Ltd.); chromatography-grade methanol (Fisher Company), chromatography-grade acetonitrile (Fisher Company), chromatography-grade formic acid (CNW Company), chromatography-grade water (Fisher Company), chromatography-grade propanol (Merck Company).

Full-wavelength multimode microplate reader Varioskan LUX, UHPLC-Orbitrap Exploris 240 (Thermo Fisher Scientific, USA); HSS T3 column (100 mm × 2.1 mm i.d., 1.8 μm, Waters Corporation, USA); JXDC-20 nitrogen blowdown concentrator (Shanghai Jingxin Industrial Development Co., Ltd., China); LNG-T88 benchtop rapid centrifugal concentrator and dryer (Taicang Huamei Biochemical Instrument Factory, China); Wonbio-96c high-throughput tissue grinder (Shanghai Wanbo Biotechnology Co., Ltd., China); Tongue electronic tongue (Shanghai Baosheng Industrial Development Co., Ltd., China).

### 2.2 Safety evaluation of functional strains

Hemolytic activity was evaluated by streaking each strain onto Columbia blood agar plates supplemented with 5% (*w*/*v*) sheep blood, followed by incubation at 37 °C for 48 h. *Staphylococcus aureus* was used as a positive control. The appearance of a greenish zone around colonies was defined as α-hemolysis, whereas a clear transparent zone was defined as β-hemolysis(Chen et al., 2024).Antibiotic susceptibility was determined using the Kirby–Bauer (K—B) disk diffusion method(Nami et al., 2014).Ten antibiotics were tested, including ampicillin, streptomycin, amikacin, kanamycin, gentamicin, penicillin, tetracycline, vancomycin, lincomycin and erythromycinand. The diameter of the inhibition zones was measured and interpreted according to CLSI guidelines: ≥20 mm was classified as sensitive (S), 10–20 mm as intermediate (I), and ≤ 10 mm as resistant (R)(Humphries et al., 2021).

### 2.3 Experimental grouping and sample preparation

Ripe and undamaged *A. squamosa* fruits were selected and washed thoroughly under running water to remove surface impurities. The fruits were manually peeled and deseeded, and the pulp was collected. The pulp was mixed with distilled water at a ratio of 3:7 (*w*/*v*) and homogenized for 2 min using a juice blender. The mixture was then filtered through double-layer sterile gauze to remove coarse particles.

The obtained juice was pasteurized at 65 °C for 30 min and rapidly cooled to room temperature. To prevent enzymatic browning and its adverse effects on fermentation, l-ascorbic acid (0.05%, *w*/*v*) was added(Isas et al., 2020). The treated juice was aseptically transferred into 250 mL sterile Erlenmeyer flasks (100 mL per flask) and stored at 4 °C until further use. Sterility was confirmed by plate counting prior to inoculation. *B. subtilis*, *A. tropicalis*, *L. reuteri*, and L. *buchneri* were activated in MRS broth at 37 °C for 24 h. Cell density was adjusted to OD600 = 1.0 using a UV–visible spectrophotometer to obtain standardized inocula. Based on preliminary experiments, each strain was inoculated into *A. squamosa* juice at an inoculum level of 5% (*v*/v)(Mustafa et al., 2019), with an initial cell density of approximately 1.0 × 10^7^ CFU/mL for all four microorganisms. Fermentation was performed at 37 °C for 48 h under shaking conditions (150 rpm). No specific aerobic or anaerobic treatments were applied; instead, passive oxygen exchange was achieved through breathable sealing during fermentation. All fermentation parameters are summarized in Supplementary Table S1. Samples were collected at 0, 4, 8, 16, 24, 36, and 48 h for subsequent physicochemical analyses, functional activity evaluations, and metabolomic profiling. The pH values of all fermentation groups and the non-fermented control were monitored at each sampling point using a calibrated pH meter. The uninoculated *A. squamosa* juice was used as the control group and designated as Nfa. Specifically, sterilized juice from the same batch was cooled to room temperature without microbial inoculation, while all other processing conditions (37 °C and 150 rpm) were maintained identically to those applied to the fermentation groups. This design minimized differences between fermentation and control groups except for microbial inoculation. The treatment groups for inoculation were named as follows: *B. subtilis* group (Bac), *A. tropicalis* group (Ace), *L. reuteri* group (Lim), and L. *buchneri* group (Lac). All fermentation experiments were conducted with three biological replicates.

### 2.4 Changes in sample composition before and after fermentation

#### 2.4.1 pH and brix measurement

During fermentation, samples were collected at 0, 4, 8, 16, 24, 36, and 48 h. Samples were centrifuged at 4500 ×*g* for 10 min, and the pH of the supernatant was immediately measured using a calibrated pH meter. Soluble solids content was determined using a digital refractometer and expressed as °Brix.

#### 2.4.2 Determination of total phenolics, total flavonoids, and free amino acids

The total phenolic content was determined using the Folin–Ciocalteu method(Dikmetas et al., 2025). Under alkaline conditions, phenolic compounds reduce phosphomolybdic–phosphotungstic acid complexes to form a blue chromophore, which was measured at 760 nm. Total flavonoid content was determined using the NaNO₂–Al(NO₃)₃–NaOH colorimetric method(Chang et al., 2020). Flavonoids form a stable red complex with aluminum ions under alkaline conditions, showing maximum absorbance at 510 nm. Free amino acid content was determined using the ninhydrin method. Amino acids react with ninhydrin under acidic and heated conditions to form Ruhemann's purple complex(Wang et al., 2022), which exhibits maximum absorbance at 570 nm, allowing quantification of total free amino acids.

(1) $$\text{Total Phenolic Content}\;\left(\mathrm{\mu g}/\mathrm{ml}\right)=286.2\times \left(A_{\mathrm{measurement}}-A_{\mathrm{blank}}+0.0006\right)\times D$$

(2) $$\text{Total Flavonoid Content}\;\left(\mathrm{mg}/\mathrm{ml}\right)=0.6\times \left(A_{\mathrm{measurement}}-A_{\mathrm{blank}}+0.0049\right)\times D$$

(3) $$\text{Amino Acid Content}\;\left(\mathrm{\mu g}/\mathrm{ml}\right)=330.6\times \left(A_{\mathrm{measurement}}-A_{\mathrm{blank}}-0.006\right)\times D$$

### 2.5 Determination of antioxidant capacity and in vitro functional activities of fermented samples

#### 2.5.1 Antioxidant activity assays

The ferric reducing antioxidant power (FRAP) was evaluated according to a previously described method(Chen et al., 2010). Antioxidants reduce the Fe^3+^–TPTZ complex to the blue-colored Fe^2+^–TPTZ form, and absorbance was measured at 593 nm. Hydroxyl radical scavenging activity was determined using a Fenton reaction system with salicylic acid as the trapping agent(Smirnoff & Cumbes, 1989). The reaction between hydroxyl radicals and salicylic acid generates a product with characteristic absorbance at 510 nm. The DPPH radical scavenging activity was measured based on the reduction of DPPH radicals, which exhibit a strong absorption at 517 nm in ethanol solution(Rubab et al., 2022).A 96-well microplate method was used for quantitative analysis. The ABTS radical scavenging activity was determined using the ABTS assay(Re et al., 1999). ABTS• + are generated by oxidation of ABTS, which display a blue-green color. Antioxidants inhibit radical formation, and the decrease in absorbance at 734 nm was recorded. All antioxidant assays were performed using commercial kits according to the manufacturer's instructions.

(4) $$\text{Free radical scavenging rate}\left(\%\right)=\left[\left(1-\frac{A_{\mathrm{measurement}}-A_{\mathrm{control}}}{A_{\mathrm{blank}}}\right)\times 100\right]\%$$

(5) $$\text{Total antioxidant capacity}\left(\mathrm{\mu mol Trolox}/\mathrm{mL}\right)=2.06\times \left(A_{\mathrm{measurement}}-A_{\mathrm{blank}}-0.0042\right)\times D$$

(6) $$\text{Hydroxyl radical scavenging rate}\;\left(\%\right)=\frac{\left[A_{\mathrm{blank}}-\left(A_{\mathrm{measurement}}-A_{\mathrm{control}}\right)\right]}{A_{\mathrm{blank}}}\times 100\%$$

(7) $$\text{DPPH radical scavenging capacity}=\left(\mathrm{\mu g}\;\mathrm{Trolox}/\mathrm{mL}\right)=0.351\times \left(\text{scavenging rate}-0.7084\right)\times D$$

(8) $$\text{ABTS radical scavenging capacity}\left(\mathrm{\mu g}\;\mathrm{Trolox}/\mathrm{mL}\right)=1.983\times \left(\mathrm{scavenging rate}+1.4213\right)\times D$$

#### 2.5.2 In vitro functional enzyme inhibitory activities

The inhibitory activities against α-amylase, α-glucosidase, pancreatic lipase, and xanthine oxidase were evaluated according to Lankatillake et al.(Lankatillake et al., 2021)with minor modifications. All assays were performed in triplicate with appropriate sample, control, and blank groups. α-Amylase inhibition was determined using soluble starch as substrate and porcine pancreatic α-amylase as enzyme source. After preincubation of sample and enzyme, the reaction was initiated by substrate addition and terminated using DNS reagent. Absorbance was measured at 540 nm. α-Glucosidase inhibition was evaluated using *p*-nitrophenyl-α-D-glucopyranoside (pNPG) as substrate and yeast α-glucosidase as enzyme. The release of p-nitrophenol was measured at 405 nm. Acarbose was used as a positive control in the α-amylase and α-glucosidase inhibition assays, while a reaction system without the test sample served as the negative control. Pancreatic lipase inhibition was assessed using *p*-nitrophenyl palmitate (pNPP) as substrate with porcine pancreatic lipase. Orlistat was used as the positive control and absorbance was measured at 405 nm. Xanthine oxidase inhibition was determined using xanthine as substrate. Uric acid formation was monitored at 295 nm. All assays included sample, sample blank, control, and blank groups. Phosphate buffer (200 mM, pH 7.4) was used as negative control, and allopurinol served as positive control.

Fermentation supernatants were obtained by centrifugation (5000 rpm, 10 min) at different time points. Protein denaturation inhibition was evaluated using egg albumin as model protein(Belle Ebanda Kedi et al., 2018). Briefly, 500 μL sample was mixed with 500 μL of 0.2% egg albumin solution and incubated at room temperature for 10 min, followed by heating at 75 °C for 10 min. After cooling and centrifugation, absorbance was measured at 660 nm. PBS was used as negative control.Fe^2+^ chelating ability was determined using the ferrozine method with minor modifications(Carter, 1971; Stookey, 1970). In a 96-well plate, 50 μL sample, 50 μL deionized water, and 50 μL FeCl₂ (2 mM) were mixed, followed by addition of 50 μL ferrozine (5 mM). After 10 min incubation at room temperature, absorbance was measured at 562 nm. A sample blank was included.

(9) $$1\;\text{Inhibition rate}\left(\%\right)=\left(1-\frac{A_{\mathrm{sample}}-A_{\mathrm{blank}}}{A_{\mathrm{control}}}\right)\times 100\%$$

(10) $$2\;\text{Inhibition rate}\left(\%\right)=\left[\frac{\left(A_{\mathrm{sample}}-A_{\mathrm{sample blank}}\right)}{\left(A_{\mathrm{control}}-A_{\mathrm{sample blank}}\right)}\right]\times 100\%$$

All experiments involving pathogenic bacteria were performed in a Biosafety Level 2 (BSL-2) laboratory in compliance with institutional biosafety guidelines. Fermentation broth after 24 h was centrifuged, and the supernatant was filtered through a 0.22 μm membrane to obtain sterile fermentation filtrate. Six foodborne pathogenic bacterial species were selected as indicator strains (all purchased from Guangdong Huankai Microbial Technology Co., Ltd.), namely *Staphylococcus aureus* (*S. aureus* ATCC25923), *Escherichia coli* (*E. coli* ATCC25922), *Pseudomonas aeruginosa* (*P. aeruginosa* GDMCC1.2476), *Salmonella enteritidis* (*S. enteritidis* GDMCC1.3028), *Shigella flexneri* (*S. flexneri* CMCC(B)51572) and *Listeria monocytogenes* (*L. monocytogenes* ATCC19115). Antimicrobial activity was evaluated using the agar well diffusion method (Moran et al., 1988; Zen et al., 2023).Bacterial suspensions were adjusted to 0.5 McFarland standard (∼1.5 × 10^8^ CFU/mL) and uniformly spread onto agar plates. Sterile Oxford cups (6 mm diameter) were placed on the agar, and 200 μL of fermentation supernatant was added into each well. Sterile water served as the negative control, ampicillin and gentamicin (10 μg/disc) were used as positive controls. Ampicillin served as a broad-spectrum positive control for all tested bacterial strains except *Pseudomonas aeruginosa*, which is intrinsically resistant to ampicillin; therefore, gentamicin was employed as the positive control for this strain. Plates were incubated at 37 °C for 24 h, and inhibition zone diameters were measured. All assays were performed in triplicate, and results were expressed as mean ± standard deviation.

### 2.6 Determination of taste

Fermented juice samples after 24 h fermentation were centrifuged, and 90 mL supernatant was collected and equally distributed into three electronic tongue sample cups, with nine biological replicates in total. Taste measurements were performed at room temperature following a standardized procedure: cleaning → conditioning → sample measurement (taste values) → cleaning → aftertaste measurement (Hayashida et al., 2023). Each measurement cycle lasted 120 s with a data acquisition interval of 1 s and no delay time. The sensor cleaning step was performed using ultrapure water for 10 s per cycle.

### 2.7 Non-targeted metabolomics analysis of differential before and after fermentation of *A. squamosa*

#### 2.7.1 Sample preparation

The fermentation broth of *A. squamosa* juice after 24 h was centrifuged, and 100 μL of the supernatant was collected into a 1.5 mL microcentrifuge tube with 400 μL solution (acetonitrile: methanol = 1:1(v:v)) containing 0.02 mg/mL internal standard (L-2-chlorophenylalanine) to extract metabolites. The samples were mixed by vortex for 30 s and low-temperature sonicated for 30 min (5 °C, 40 KHz). The samples were placed at −20 °C for 30 min to precipitate the proteins. Then the samples were centrifuged for 15 min (4 °C, 13000 g). The supernatant was removed and blown dry under nitrogen. The sample was then re-solubilized with 100 μL solution (acetonitrile: water = 1:1) and extracted by low-temperature ultrasonication for 5 min (5 °C, 40 KHz), followed by centrifugation at 13000 *g* and 4 °C for 10 min. The supernatant was transferred to sample vials for LC-MS/MS analysis. Equal aliquots from all control and experimental groups were combined to prepare quality control (QC) samples. These QC samples were inserted before, during, and after the analytical run to assess and ensure the stability, reproducibility, and reliability of the entire analytical system.

#### 2.7.2 LC-MS/MS analysis

The LC-MS/MS analysis of sample was conducted on a Thermo UHPLC-Q Exactive system equipped with an ACQUITY HSS T3 column (100 mm × 2.1 mm i.d., 1.8 μm, Waters, USA) at Majorbio Bio-Pharm Technology Co. Ltd. (Shanghai, China).The mobile phases consisted of 0.1% formic acid in water: acetonitrile (95,5, *v*/v) (solvent A) and 0.1% formic acid inacetonitrile: isopropanol: water (47.5,47.5, *v*/v)(solvent B). The flow rate was 0.40 mL/min and the column temperature was 40 °C.

MS conditions:

The UPLC system was coupled to a Thermo UHPLC-Q Exactive Mass Spectrometer equipped with an electrospray ionization (ESI) source operating in positive mode and negative mode. The optimal conditions were set as followed: source temperature at 400 °C; sheath gas flow rate at 40 arb; Aux gas flow rate at 10 arb; ion-spray voltage floating (ISVF) at -2800 V in negative mode and 3500 V in positive mode, respectively; Normalized collision energy, 20–40-60 V rolling for MS/MS. Full MS resolution was 70,000, and MS/MS resolution was 17,500. Data acquisition was performed with the Data Dependent Acquisition (DDA) mode. The detection was carried out over a mass range of 70–1050 *m*/*z*.de.

#### 2.7.3 Statistical analysis

All experiments were performed with three biological replicates (*n* = 3), and results are presented as mean ± standard deviation (SD). Statistical analyses were conducted using GraphPad Prism 9.5 (version 9.5). Differences among groups were analyzed by one-way analysis of variance (ANOVA), followed by Tukey's HSD test for multiple comparisons among fermentation groups and Dunnett's test for comparisons with the non-fermented control. A two-tailed *p* < 0.05 was considered statistically significant.

LC–MS data were processed using Progenesis QI (Waters Corporation, Milford, MA, USA) for peak detection, alignment, normalization, and metabolite annotation. Metabolites were putatively identified by matching accurate mass, retention time, isotope distribution, and MS/MS spectra against the KEGG, HMDB, METLIN, and LipidBlast databases (MSI Level 2). Peak intensities were normalized using the 80% rule combined with sum normalization, and variables with QC relative standard deviation (RSD) > 30% were excluded. PCA and OPLS-DA were performed using the **ropls** package (version 1.6.2) in R, and model robustness was evaluated by seven-fold cross-validation. Differential metabolites were identified using the criteria of VIP > 1.0, FC ≥ 1.5 or ≤ 0.67, and FDR-adjusted *p* < 0.05. KEGG pathway enrichment analysis was performed using a hypergeometric test with Benjamini–Hochberg correction. Pearson correlation analysis was used to evaluate relationships between differential metabolites and bioactivity parameters, with *p* < 0.05 considered statistically significant. Correlation heatmaps and KEGG pathway enrichment plots were generated using R and Python, respectively.

## 3 Results and discussion

### 3.1 Safety evaluation of functional strains

**Fig. 1:**
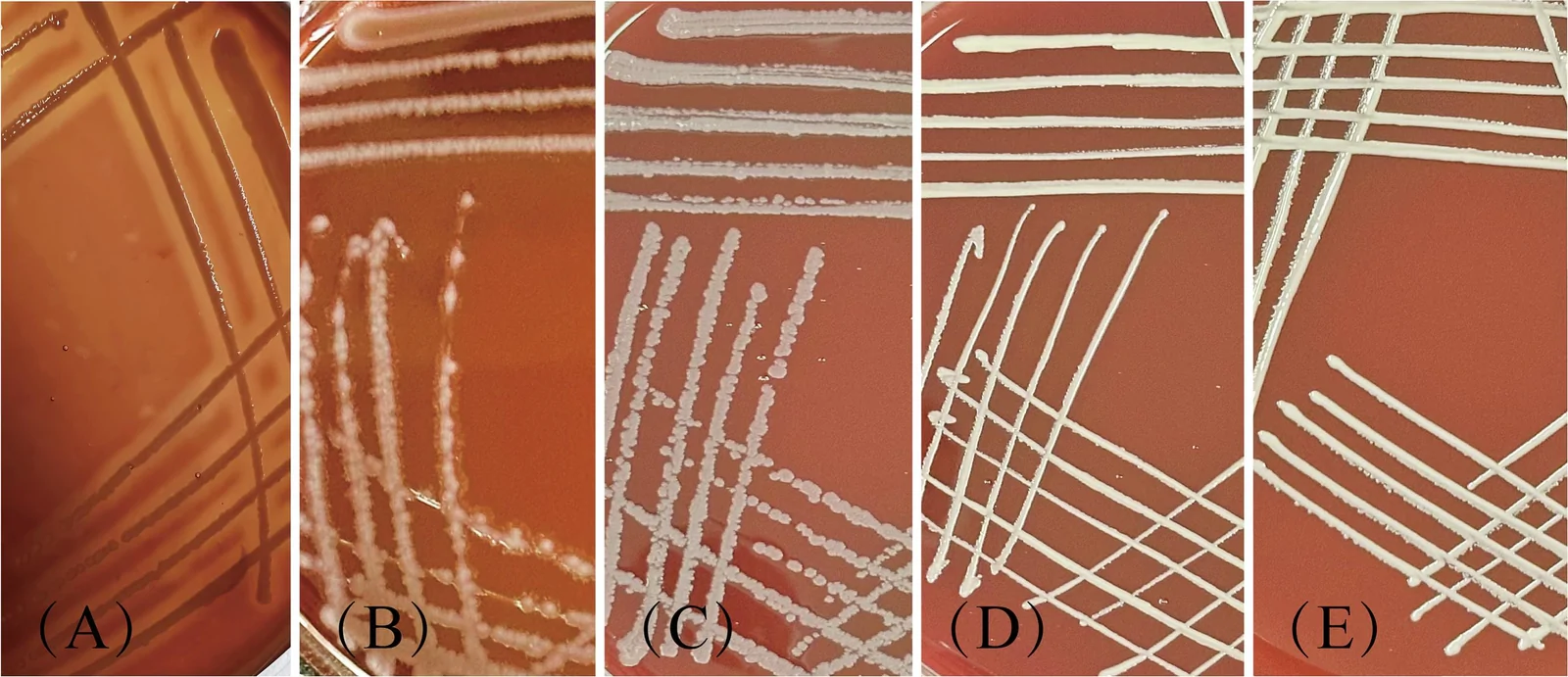
Hemolytic activity of functional strains. Representative hemolysis assay plates of (A) *Staphylococcus aureus*, (B) *Bacillus subtilis*, (C) *Acetobacter tropicalis*, (D) *Limosilactobacillus reuteri*, and (E) *Lactobacillus buchneri*.

As shown in Fig. 1, compared with the positive control (*Staphylococcus aureus*, Fig. 1A), no obvious hemolytic zones were observed for *B. subtilis* (Fig. 1B), *A. tropicalis* (Fig. 1C), *L. reuteri* (Fig. 1D), and L. *buchneri* (Fig. 1E), indicating that all tested strains exhibited no detectable hemolytic activity or only negligible hemolysis. These results support their suitability for food fermentation and confirm they are safe for food-related applications. As summarized in Table 1, the four strains exhibited differential sensitivity patterns to the tested antibiotics, while overall compliance with food-grade safety requirements was observed. The positive control strain (*Staphylococcus aureus*) exhibited the expected susceptibility profile, confirming the reliability of the assay. *B. subtilis* showed relatively high sensitivity to aminoglycoside antibiotics, including amikacin, kanamycin, and gentamicin, but resistance to penicillin. *A. tropicalis* exhibited generally high sensitivity across most tested antibiotics. In contrast, L. *reuteri* and *L. buchneri* showed intermediate or low sensitivity to certain antibiotics. Previous studies have reported that the intrinsic antibiotic resistance of lactic acid bacteria is mainly associated with cell wall structure, membrane permeability, and efflux systems, rather than the presence of transferable resistance genes. In addition, natural resistance to aminoglycosides has been attributed to the absence of a functional electron transport chain(Yan et al., 2024). Collectively, these findings indicate that the selected strains satisfy the safety parameters evaluated in this study and are suitable candidates for subsequent fermentation experiments.

**Table 1:** Antibiotic susceptibility of functional strains.

antibiotic	content	Diameter of the zone of inhibition /mm				
		Control	*B. subtilis*	*A. tropicalis*	*L. reuteri*	*L. buchneri*
Ampicillin	10 μg	9.44 ± 0.09	19.96 ± 1.57(I)	21.87 ± 0.56(S)	13.63 ± 2.08(I)	16.3 ± 0.98(I)
Streptomycin	10 μg	18.37 ± 0.32(I)	16.7 ± 1.04(I)	14.89 ± 0.6(I)	–	–
Amikacin	30 μg	8.72 ± 0.34	21.48 ± 0.70(S)	31.2 ± 1.06(S)	11.36 ± 0.09(I)	11.64 ± 0.69(I)
Kanamycin	30 μg	14.28 ± 1.23(I)	24.75 ± 1.96(S)	32.33 ± 0.97(S)	–	–
Gentamicin	10 μg	9.53 ± 0.26	31.33 ± 1.03(S)	34.33 ± 3.11(S)	14.68 ± 0.47(I)	14.25 ± 0.06(I)
Penicillin	10 U	18.24 ± 0.62(I)	8.37 ± 0.24	24.46 ± 0.77(S)	17.43 ± 2.65(S)	18.91 ± 0.15(I)
Tetracycline	30 μg	6.82 ± 0.22	15.68 ± 3.59(I)	15.74 ± 0.6(I)	11.32 ± 0.9(I)	15.05 ± 0.27(I)
Vancomycin	30 μg	6.89 ± 0.39	21.22 ± 0.86(S)	23.92 ± 2.99(S)	12.18 ± 0.04	18.42 ± 0.3(I)
Lincomycin	2 μg	14.85 ± 0.61(I)	16.34 ± 0.98(I)	18.97 ± 0.72(I)	11.58 ± 2.21(I)	–
Erythromycin	15 μg	8.29 ± 0.30	25 ± 1.48(S)	31.04 ± 1.14(S)	17.26 ± 0.18(I)	11.73 ± 0.35(I)

Note: Values are expressed as the mean ± SD of three independent experiments. Antibiotic susceptibility was classified according to the CLSI guidelines as follows: susceptible (S), inhibition zone diameter ≥ 20 mm; intermediate (I), 10–20 mm; and resistant (R), ≤ 10 mm.

### 3.2 The effects of fermentation on bioactive compounds in *A. squamosa* juice

#### 3.2.1 Changes in pH and brix

**Fig. 2:**
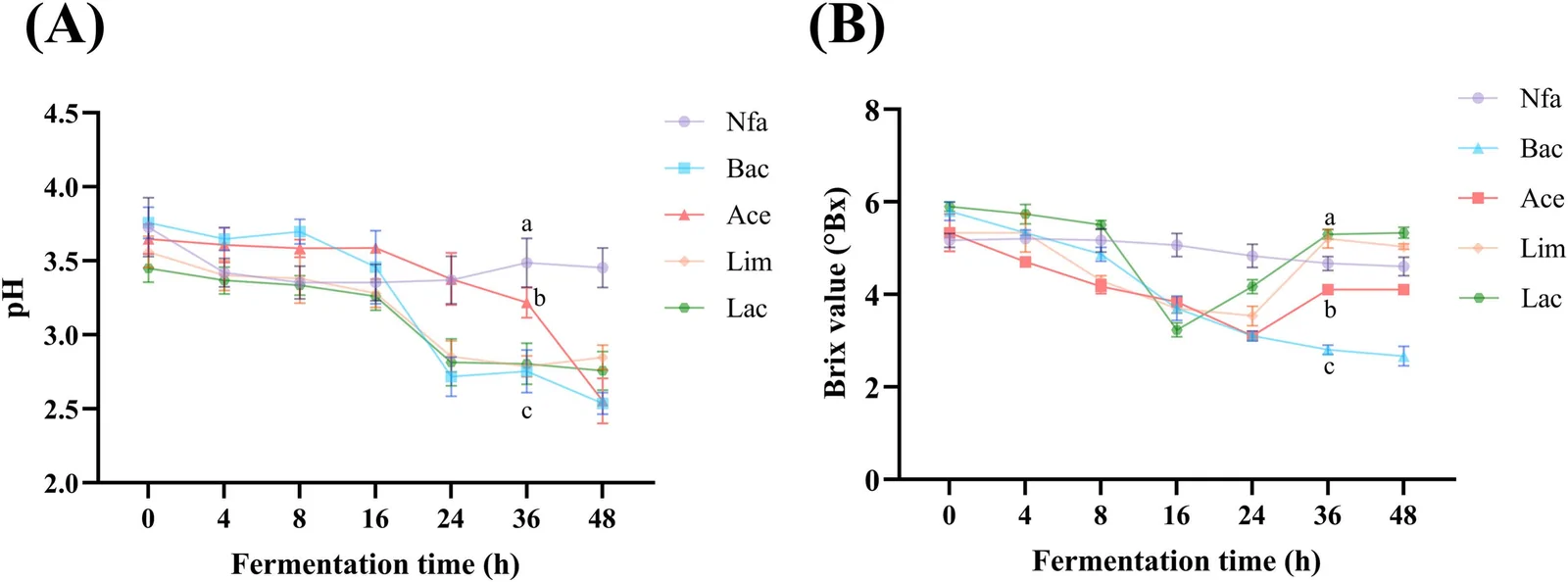
Changes in pH and Brix during fermentation by functional strains(A) pH of the fermentation broth(B) Brix of the fermentation broth.

Changes in pH during fermentation are shown in Fig. 2A. All fermentation systems exhibited a continuous decreasing trend. Notably, the Lim and Lac groups showed a rapid decline within the first 16 h, indicating strong early acidification capacity. In contrast, the Bac and Ace groups exhibited a more pronounced pH decrease during the mid-to-late fermentation stage. The decrease in pH was mainly attributed to microbial carbohydrate metabolism and the accumulation of organic acids, particularly lactic and acetic acids, during fermentation(Filannino et al., 2017). As shown in Fig. 2B, total soluble solids (°Brix) decreased significantly in all fermentation groups. The Bac group showed the most pronounced and continuous decline, whereas Lim, Lac, and Ace exhibited a stabilization or slight rebound after 24 h. This phenomenon may be associated with differences in carbon utilization strategies among strains and the release of soluble polysaccharides or oligosaccharides during late fermentation stages. The greater decrease observed in the Bac group may reflect the stronger carbohydrate utilization capacity of *B. subtilis*, resulting in continuous sugar consumption during fermentation(Papagianni, 2012). pH reduction and °Brix decline showed a coupled relationship, reflecting microbial carbon metabolism intensity during fermentation(Marco et al., 2016; Mercade et al., 2000).

#### 3.2.2 Total phenolics, total flavonoids, and free amino acids

**Fig. 3:**
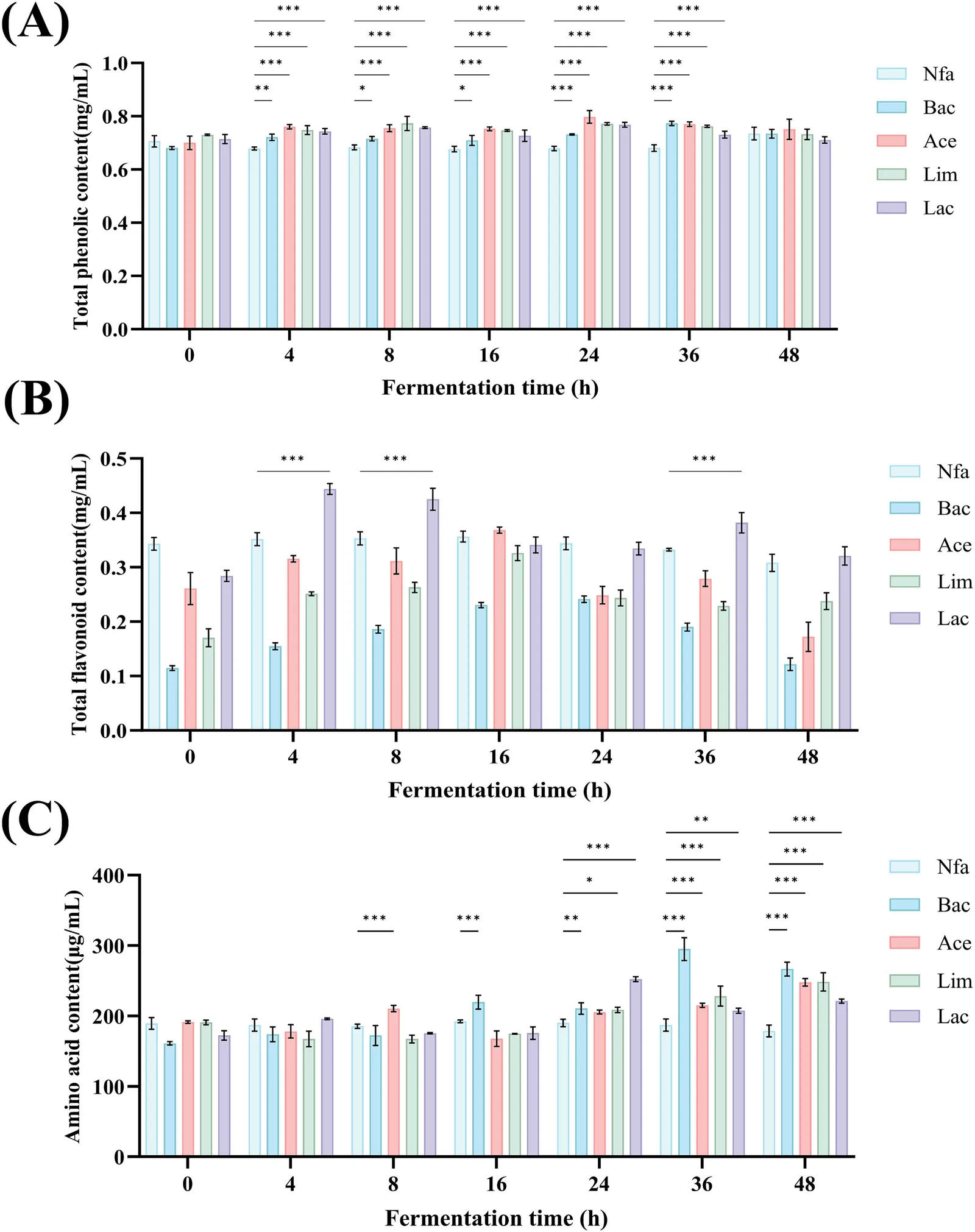
Changes in total phenolic content (A), total flavonoid content (B), and free amino acid content (C) during fermentation by different functional strains.

As shown in Fig. 3A, total phenolic content increased significantly in all fermentation systems during the early and middle stages (*P* < 0.001), followed by a plateau at the late fermentation stage. The control group showed only minor variation. This increase is mainly associated with microbial enzymatic activities (e.g., esterases and polyphenol-related enzymes), which promote the release of bound phenolics. In addition, acidification during fermentation facilitates cell wall degradation and enhances phenolic liberation(Hur et al., 2014). Total flavonoid content (Fig. 3B) remained stable in the control group, whereas the Lac group showed a significant increase from 0.28 ± 0.01 to 0.43 ± 0.02 mg/mL (*P* < 0.001). This may be due to glycosidase-mediated hydrolysis converting flavonoid glycosides into free aglycones, combined with structural disruption of plant tissues under acidic conditions(Rodríguez et al., 2009). Other strains showed an initial increase followed by stabilization, suggesting a balance between release and oxidative transformation of flavonoids. Free amino acid content (Fig. 3C) increased significantly in all inoculated groups at the later fermentation stage (*P* < 0.001), indicating strong proteolytic and peptidolytic activities. This is attributed to microbial secretion of proteases and peptidases, which hydrolyze proteins and peptides into free amino acids(Pessione, 2012). The control group showed only slight changes. These amino acid shifts may further influence flavor development, particularly umami-related compounds such as glutamic acid and aspartic acid.

### 3.3 In vitro activity of fermentation broth

#### 3.3.1 In vitro antioxidant activity of fermented juice

**Fig. 4:**
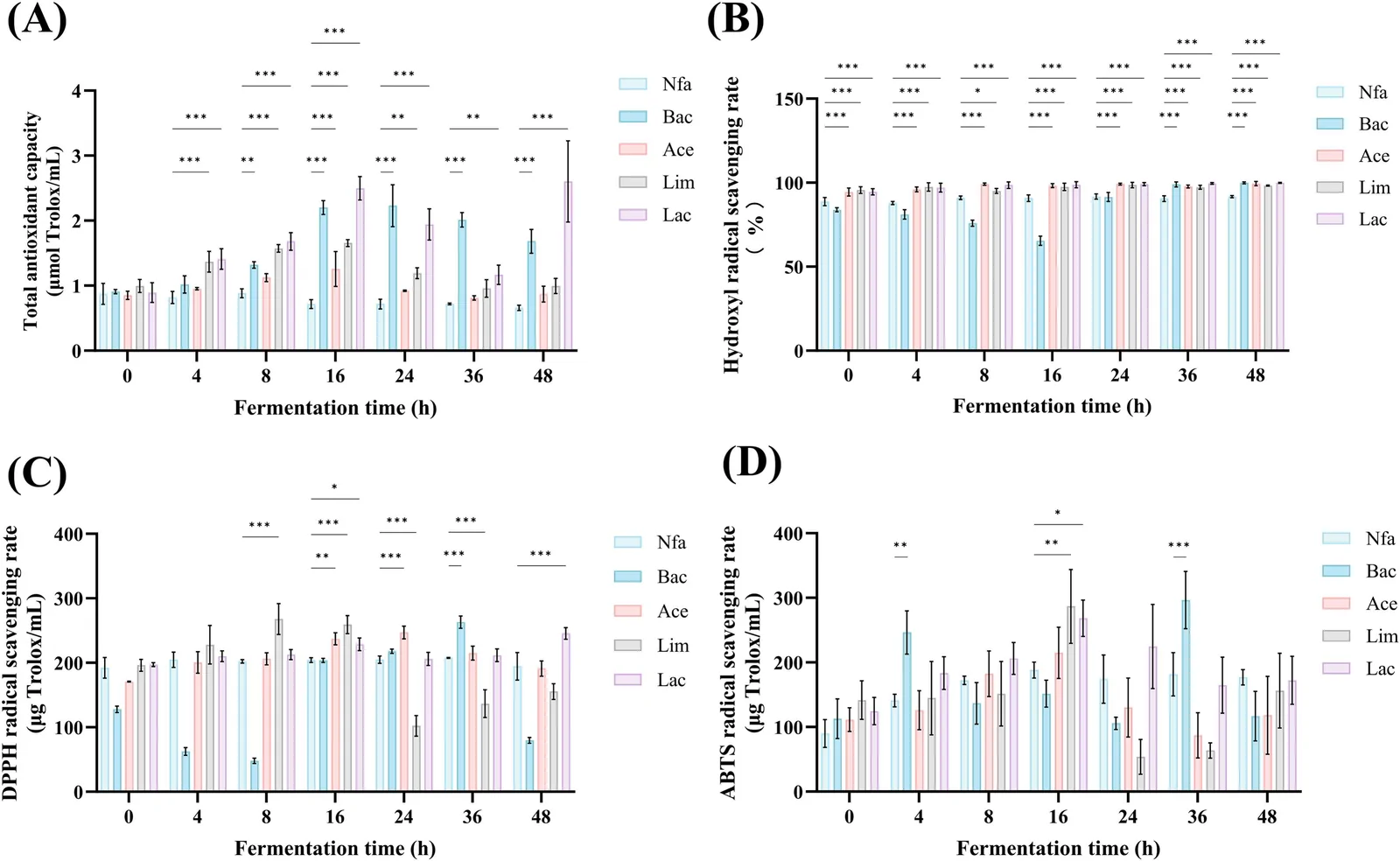
Antioxidant capacity of the fermentation broth. (A) Total antioxidant capacity, (B) Hydroxyl radical scavenging rate, (C) DPPH radical scavenging capacity, (D) ABTS radical scavenging capacity.

Antioxidant activities were evaluated using FRAP, hydroxyl radical, ABTS^+^ and DPPH assays. FRAP values (Fig. 4A) increased significantly during the first 8–16 h (*P* < 0.001), reaching a peak at 16 h, followed by a slight decline. Bac and Lac groups showed the highest activity. This is mainly attributed to the accumulation of reducing compounds such as phenolics and flavonoids, which reduce Fe^3+^ to Fe^2+^(Marcu et al., 2025). Hydroxyl radical scavenging activity (Fig. 4B) remained relatively stable in Ace, Lim, and Lac groups, while Bac showed a fluctuation pattern. These compounds may act via hydrogen or electron donation to neutralize reactive oxygen species(Platzer et al., 2022). ABTS^+^ scavenging capacity (Fig. 4C) increased with fermentation time and peaked at different time points among groups, with Ace and Lim showing significantly higher activity than the control (*P* < 0.001). This reflects the accumulation of polar antioxidant metabolites(Re et al., 1999). DPPH scavenging activity (Fig. 4D) showed an initial increase followed by a decline, peaking at mid-fermentation. This trend is consistent with changes in phenolic and flavonoid content and may be related to further oxidation or metabolic conversion of antioxidants at later stages(Shahidi & Ambigaipalan, 2015).Overall, fermentation significantly enhanced antioxidant capacity, primarily due to the release and transformation of phenolic and flavonoid compounds.

#### 3.3.2 In vitro functional study of fermentation broth

**Fig. 5:**
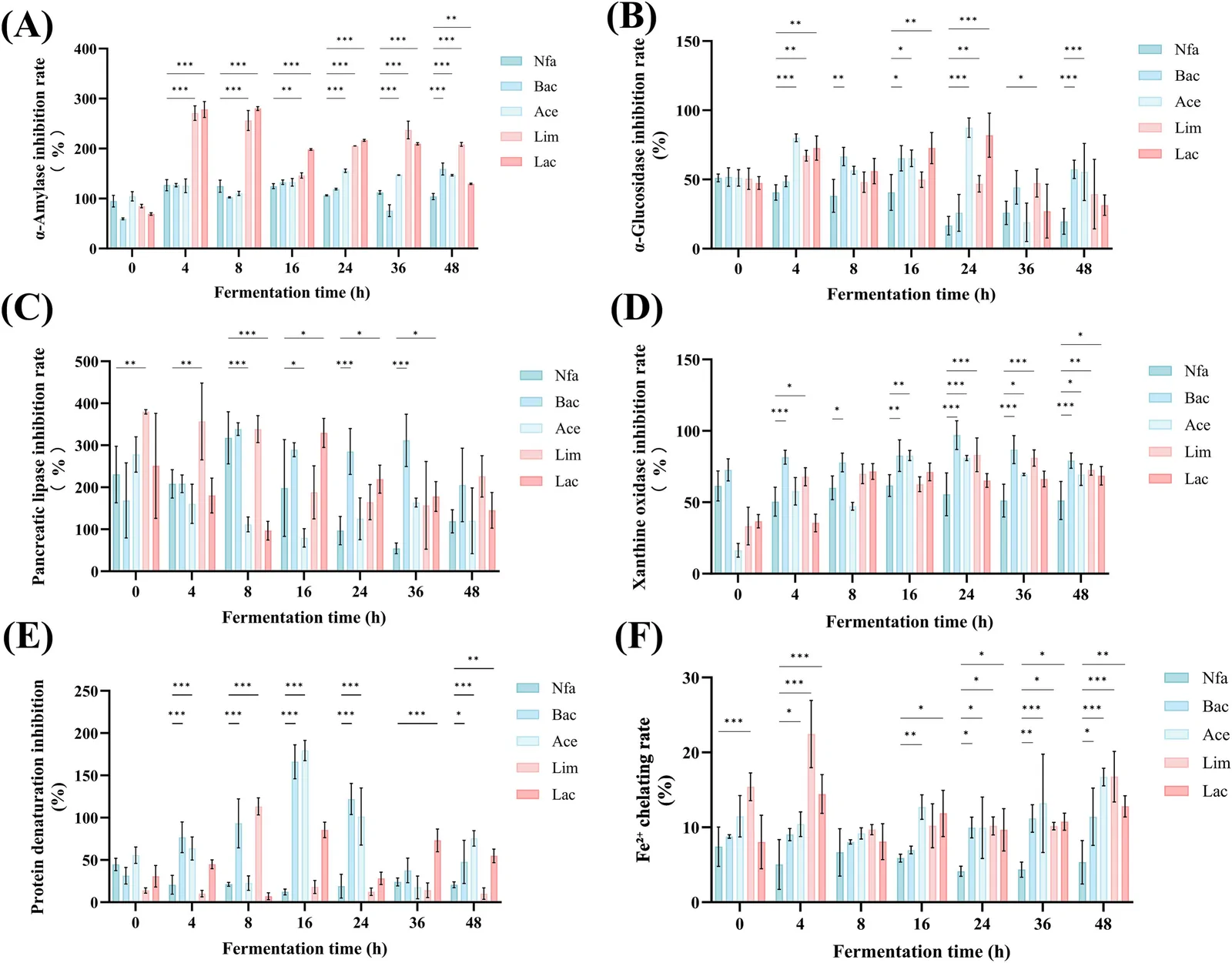
In vitro activities of fermentation broth. (A) α-Amylase inhibition, (B) α-Glucosidase inhibition, (C) Pancreatic lipase inhibition, (D) Xanthine oxidase inhibition, (E) Protein denaturation inhibition, (F) Fe^2+^ chelation.

As shown in Fig. 5A–D, fermentation significantly enhanced inhibitory activities against α-amylase, α-glucosidase, pancreatic lipase, and xanthine oxidase (XOD), although with strain-dependent differences. α-Amylase inhibition (Fig. 5A) increased in Lim and Lac groups during fermentation, while Bac and Ace showed stronger effects at later stages. This is likely related to phenolics and flavonoids interacting with enzyme active sites or altering enzyme conformation(McDougall et al., 2005). The positive control exhibited an inhibition rate of (99.10 ± 0.21)%, indicating that the enzyme reaction system was functionally active under the experimental conditions. α-Glucosidase inhibition (Fig. 5B) showed a time-dependent increase, peaking at 24 h, this value was lower than that of the positive control, which was (107.82 ± 7.47)%. especially in Ace and Lac groups (*P* < 0.001), followed by a decline, possibly due to metabolic transformation of active phenolics(Tadera et al., 2006). Pancreatic lipase inhibitory activity (Fig. 5C) exhibited strain-dependent differences, the positive control showed an inhibition rate of (95.28 ± 1.87)%, confirming that the experimental system was sensitive and reliable. The *Bac* group reached its peak at 8 h and then declined, whereas the *Ace*, *Lim*, and *Lac* groups showed an initial decrease followed by an increase, with the *Ace* group exhibiting relatively weak activity overall. This variation may be attributed to the formation of complexes between polyphenols and lipase, or the synergistic effects of small-molecular-weight peptides and organic acids generated during fermentation(He et al., 2023). Xanthine oxidase (XOD) inhibitory activity (Fig. 5D) showed a continuous increase in all four fermentation systems over time, reaching its maximum at 24 h (*P* < 0.001), the inhibition rates were close to or comparable to that of the positive control (87.07 ± 5.31)%, indicating that the experimental system was reliable and well-performing. The enhanced XOD inhibition may be associated with increased levels of antioxidant metabolites after fermentation. In conclusion, fermentation with each of the four functional bacterial strains significantly enhanced the inhibitory capacity of soursop juice against multiple metabolism-related enzymes, albeit with differences in peak time and potency among strains. These findings suggest that fermentation may enhance the in vitro enzyme inhibitory activities of *A. squamosa* juice through alterations in fermentation-derived metabolites.

Protein denaturation inhibition (Fig. 5E) peaked at 16–24 h in Bac and Ace groups, while lactic acid bacteria maintained stable activity. This may be related to phenolic and peptide-mediated stabilization of protein structure. Fe^2+^ chelating ability (Fig. 5F) showed a general increasing trend, with Lim exhibiting the strongest early activity. Chelation of Fe^2+^ may suppress Fenton reaction-mediated oxidative damage(Chung et al., 2015). Overall, fermentation enhanced anti-inflammatory potential through combined metal chelation and protein stabilization effects. It should be emphasized that these bioactivities were evaluated exclusively through in vitro assays and therefore cannot be regarded as direct evidence of in vivo efficacy.

#### 3.3.3 Analysis of the inhibitory activity of fermentation broth against foodborne pathogens

Antimicrobial activity of 24 h fermented juice is shown in Table 2. The non-inoculated control (Nfa) exhibited moderate inhibitory effects, while sterile water showed no activity, indicating that native phenolics and secondary metabolites in *A. squamosa* contribute to baseline antimicrobial properties(Baskar et al., 2007). After fermentation, antimicrobial activity was significantly enhanced. Bac group showed the strongest inhibition against *E. coli*, *S. enteritidis, and* L. *monocytogenes*, likely due to production of lipopeptides and antimicrobial peptides that disrupt bacterial membranes(Ongena & Jacques, 2008). Lim group exhibited strong inhibition against *S. aureus* and *S. enteritidis*；Lac *group* demonstrated significant inhibitory effects against L. *monocytogenes*, likely mediated by organic acids and hydrogen peroxide. Ace group showed moderate activity, mainly associated with acidification effects(Raspor & Goranovič, 2008). Bac and Lim groups demonstrated the strongest antimicrobial performance, suggesting synergistic production of organic acids, bacteriocins, antimicrobial peptides, and phenolic compounds during fermentation.

**Table 2:** Antimicrobial activity of fermented juices against foodborne pathogens and antibiotic susceptibility classification (mm).

Pathogen	Negative control	Positive control	Nfa	Bac	Ace	Lim	Lac
*S. aureus*	–	22.75 ± 1.73 (S) a	17.19 ± 0.90 (I) b	19.24 ± 0.81 (I) b	19.03 ± 1.91 (I) b	22.05 ± 2.71 (S) a	18.52 ± 1.15 (I) b
*E. coli*	–	24.05 ± 1.72 (S) a	16.87 ± 1.28 (I) b	21.33 ± 0.83 (S) a	16.56 ± 1.25 (I) b	15.82 ± 1.62 (I) b	17.20 ± 2.37 (I) b
*P. aeruginosa*	–	17.56 ± 0.76 (I) a	17.10 ± 1.19 (I) a	17.6 ± 1.57 (I) a	16.88 ± 1.99 (I) a	18.24 ± 1.83 (I) a	17.21 ± 0.90 (I) a
*S. enteritidis*	–	28.38 ± 0.86 (S) a	14.73 ± 2.12 (I) c	22.25 ± 0.92 (S) b	15.52 ± 1.29 (I) c	20.35 ± 1.38 (S) b	16.47 ± 0.79 (I) c
*S. flexneri*	–	23.86 ± 1.59 (S) a	16.80 ± 2.99 (I) b	18.82 ± 1.37 (I) b	19.51 ± 0.76 (I) b	19.86 ± 1.25 (I) b	14.72 ± 1.98 (I) b
*L. monocytogenes*	–	25.90 ± 1.36 (S) a	11.34 ± 0.95 (I) c	21.38 ± 1.05 (S) b	11.16 ± 0.58 (I) c	11.01 ± 0.37 (I) c	22.87 ± 1.20 (S) b

Note: Data are expressed as mean ± SD (*n* = 3). Values in parentheses indicate antibiotic susceptibility classification according to CLSI: S ≥ 20 mm, *I* = 10–20 mm, and *R* ≤ 10 mm. Different lowercase letters indicate significant differences (*p* < 0.05).

### 3.4 Taste profile differences of fermented *A. squamosa* juice

**Fig. 6:**
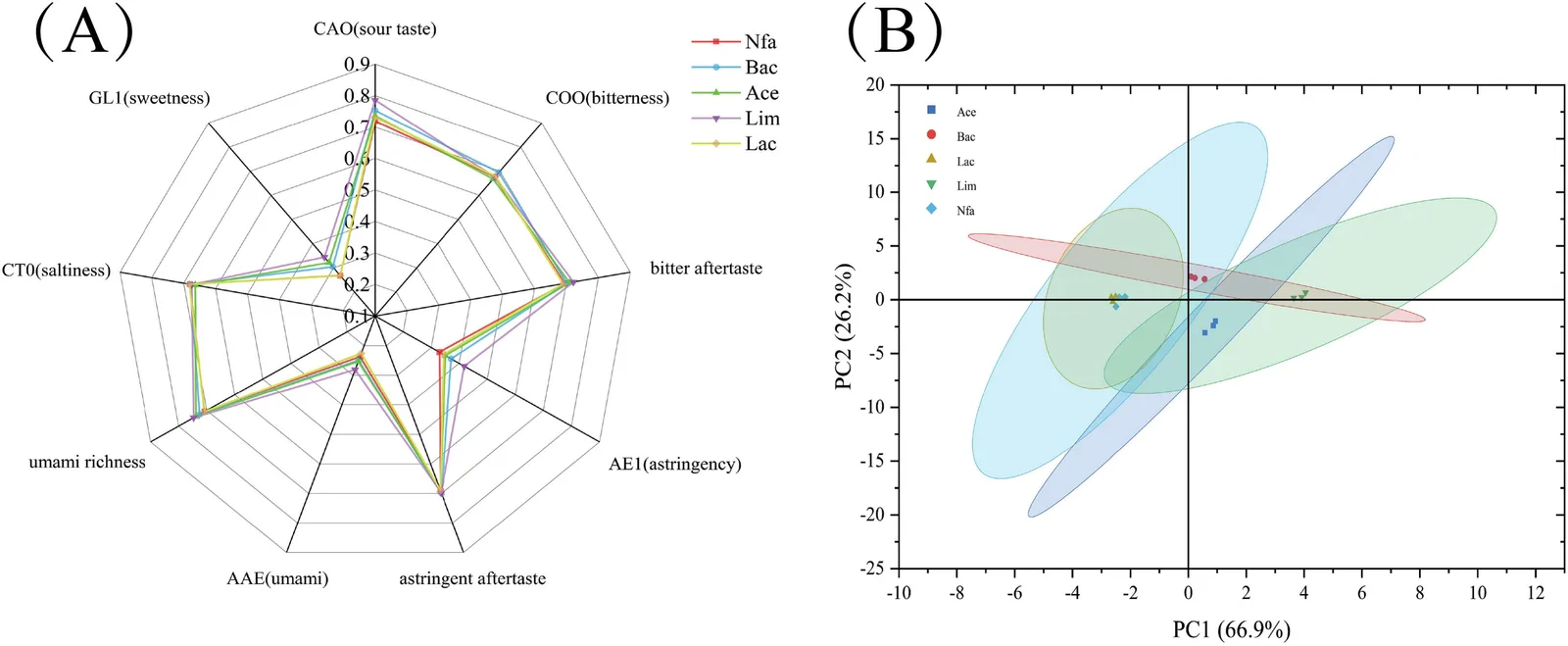
Taste analysis of *A. squamosa* fermentation broth by electronic tongue. (A) Taste radar chart, (B) Principal Component Analysis (PCA).

Electronic tongue analysis (Fig. 6A) showed that all fermented samples exhibited similar overall taste response patterns, with sourness (CAO) and bitterness (COO) as the dominant attributes, while astringency (AE1), umami (AAE), sweetness (GL1), and saltiness (CT0) were relatively low. Compared with the non-inoculated control (Nfa), fermentation increased sourness, sweetness, and umami, while reducing bitterness and astringency. The enhanced sourness was mainly associated with the accumulation of organic acids such as lactic and acetic acids, which also contributed to pH reduction and the release of taste-active compounds. Increased sweetness and umami were closely related to the generation of free amino acids and small peptides derived from protein hydrolysis, particularly glutamic and aspartic acids, key contributors to umami perception(Pessione & Cirrincione, 2016). In contrast, the decrease in bitterness and astringency may result from structural transformations of phenolic compounds and their reduced interactions with proteins. Principal component analysis (Fig. 6B) showed that PC1 and PC2 explained 66.9% and 26.2% of the total variance, respectively, with a cumulative contribution of 93.1%, indicating clear discrimination among samples. Overall, fermentation mainly modulated taste intensity through organic acid accumulation and proteolysis, while strain differences were primarily reflected in metabolic intensity.

### 3.5 Non-targeted metabolomics analysis

#### 3.5.1 Comparative analysis of fermentation broth samples

**Fig. 7:**
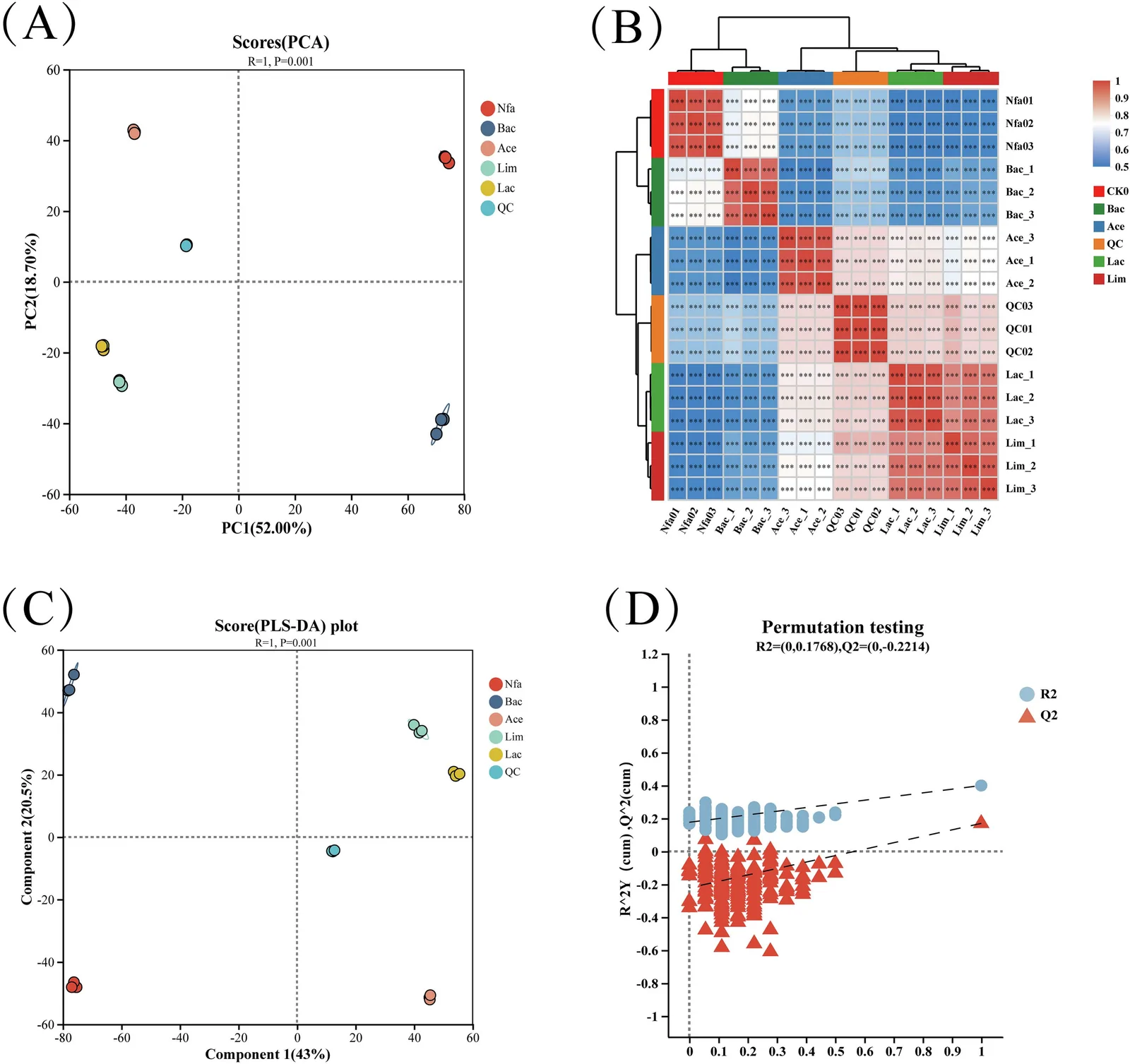
Comparative analysis of *A. squamosa* juice samples after 24 h of fermentation. (A) Principal Component Analysis (PCA), (B) Correlation heatmap, (C) Partial Least Squares Discriminant Analysis (PLS-DA), (D) PLS-DA permutation test.

Untargeted metabolomics was performed on 24 h fermented *A. squamosa* juice (four bacterial strains) and non-fermented juice (control). Sample similarity was evaluated using correlation heatmaps, while principal component analysis (PCA) and partial least squares-discriminant analysis (PLS-DA) were used to assess metabolic differences.As shown in Fig. 7A, PCA explained 52.0% (PC1) and 18.7% (PC2) of the total variance. Clear separation between fermented and non-fermented groups was observed, indicating that fermentation significantly reshaped the overall metabolic profile. Replicate samples clustered closely, demonstrating good reproducibility.The correlation heatmap (Fig. 7B) showed high intra-group similarity, with correlation coefficients close to 1, confirming strong consistency among biological replicates. In contrast, clear inter-group differences were observed, indicating strain-dependent modulation of metabolite composition.PLS-DA (Fig. 7C) further improved group separation, indicating distinct metabolic patterns among fermentation groups. The permutation test yielded R^2^ = 0.1768 and Q^2^ = −0.2214, suggesting that the PLS-DA model did not show evidence of significant overfitting and had limited predictive performance.

#### 3.5.2 Statistical analysis of fermentation broth metabolites

**Fig. 8:**
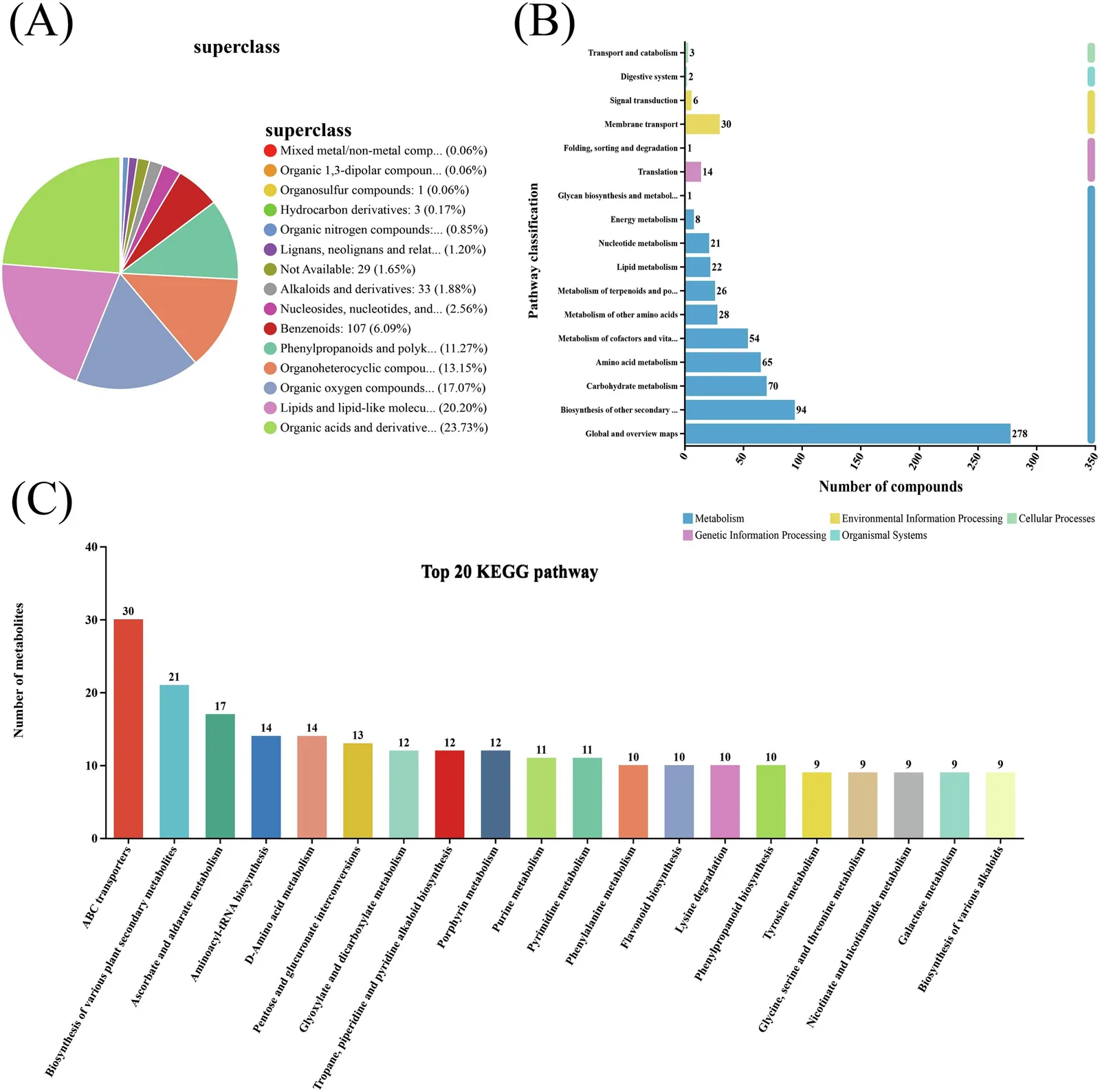
Metabolite statistics of *A. squamosa* fermentation broth. (A) Classification of HMDB compounds, (B) Pathways associated with metabolic regulation, (C) Top 20 enriched metabolic pathways.

A total of 12,563 positive-ion and 8995 negative-ion features were detected. Metabolite annotation based on KEGG and HMDB databases resulted in the annotation of 686 and 1757 metabolite features, respectively. Metabolite classification (Fig. 8A) revealed that organic acids and derivatives, lipids, and lipid-like molecules were the dominant classes, including amino acids, peptides, fatty acids, and glycerophospholipids. Other major categories included carbohydrates, organic heterocyclic compounds, phenylpropanoids, polyketides, flavonoids, and glycosides. These distributions indicate active carbohydrate degradation and lipid metabolism during fermentation. Pathway annotation (Fig. 8B) showed that metabolites were mainly involved in secondary metabolite biosynthesis, carbohydrate metabolism, amino acid metabolism, cofactor and vitamin metabolism, lipid metabolism, terpenoid and polyketide biosynthesis, and other amino acid-related pathways. Top 20 KEGG pathway enrichment (Fig. 8C) highlighted significant involvement of ABC transporters, biosynthesis of secondary metabolites, ascorbate and aldarate metabolism, aminoacyl-tRNA biosynthesis, D-amino acid metabolism, and flavonoid biosynthesis. Amino acid-related pathways were among the major enriched pathways, suggesting substantial metabolic remodeling during fermentation.

#### 3.5.3 Differential metabolite analysis

**Fig. 9:**
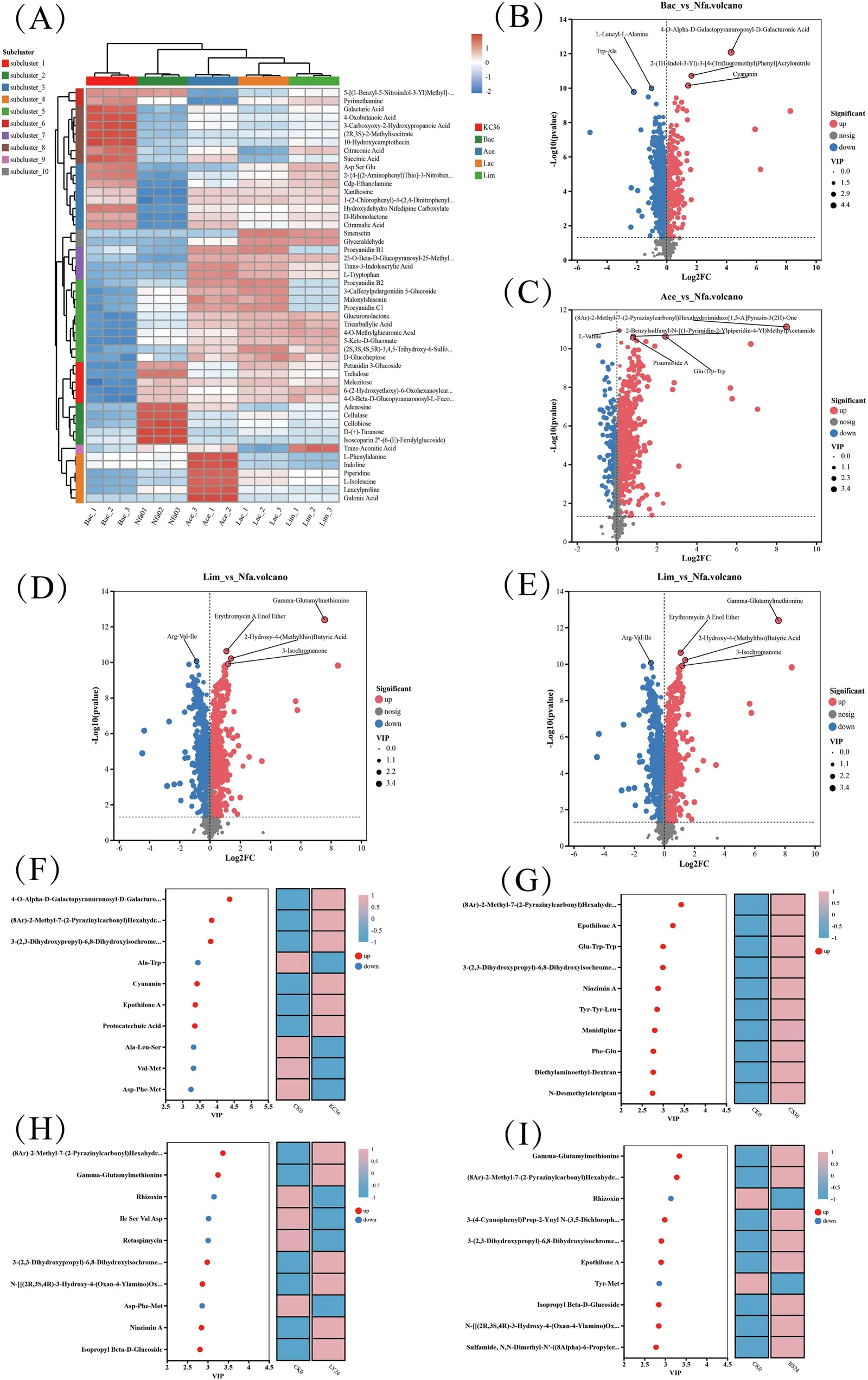
Differential metabolite analysis of *A. squamosa* fermentation broth. (A) Hierarchical clustering heatmap of differential metabolites in fermentation broth, (B-E) Volcano plot of differential metabolites, (F—I) VIP score plot.

Hierarchical clustering of the top 50 metabolites (Fig. 9A) showed that biological replicates clustered within the same group, confirming high data reliability and reproducibility. Distinct metabolite abundance patterns were observed among different treatment groups, indicating strong strain-specific metabolic regulation. Volcano plots (Fig. 9B–E) revealed a large number of significantly up- and down-regulated metabolites in all fermentation groups compared with the control. Importantly, different strains induced distinct metabolic shifts. In the Bac vs Nfa group (Fig. 9B), metabolites such as cyananin and protocatechuic acid were significantly upregulated, while several amino acid- and dipeptide-related metabolites (e.g., leucyl-alanine and tryptophan-containing dipeptides) were downregulated, likely due to enhanced proteolysis and peptide metabolism by *B. subtilis*. In the Ace vs Nfa group (Fig. 9C), metabolites such as L-valine and tryptophan-related peptides were significantly altered, suggesting modulation of amino acid metabolism under oxidative fermentation conditions. In the Lim vs Nfa group (Fig. 9D), γ-glutamylmethionine was significantly increased, indicating enhanced glutamyl cycle activity and amino acid transport in L. *reuteri* fermentation. In the Lac vs Nfa group (Fig. 9E), both amino acid-derived peptides and phenolic glycosides (e.g., arginine-proline dipeptide and malvidin-3-caffeoyl-glucoside) were significantly upregulated, suggesting simultaneous protein hydrolysis and phenolic release during lactic acid fermentation.

Statistical analysis was performed on the top 10 significantly differential metabolites between the fermentation groups and the control group. In the Bac group (Fig. 9F), metabolites such as cyanidin, epothilone A, and protocatechuic acid were significantly upregulated. In the Ace group (Fig. 9G), epothilone A, manidipine, tyr-tyr-leu, **phe-gln**, and *N*-desmethyllexitriptan showed significant upregulation. The Lim group (Fig. 9H) was enriched in γ-glutamylmethionine, niaziminin A, and isopropyl-β-D-glucoside. Meanwhile, the Lac group (Fig. 9I) exhibited significant increases in epothilone A, γ-glutamylmethionine, and isopropyl-β-D-glucoside. Notably, among the upregulated metabolites, cyanidin and protocatechuic acid are well-recognized antioxidants with free radical scavenging and anti-inflammatory activities(Kim et al., 2025; Ockermann et al., 2021). Interestingly, epothilone A was significantly upregulated in multiple fermentation groups. As a microtubule-stabilizing compound similar to paclitaxel(Sepp-Lorenzino et al., 2002) (Bollag et al., 1995),it exhibits strong antiproliferative activity and potential anticancer properties (Prota et al., 2013) γ-Glutamylmethionine is a key intermediate in glutathione metabolism and contributes to oxidative stress protection(Townsend et al., 2003). Niaziminin A, a glucosinolate derivative, has been reported to exhibit antihypertensive activity(Faizi et al., 1994; Khan et al., 2019). Alkyl glucosides such as isopropyl-β-D-glucoside may influence membrane permeability and microbial interactions(Céliz et al., 2013).These findings suggest that different microbial strains drive the accumulation of diverse bioactive metabolites through distinct metabolic pathways, contributing to the functional properties of fermented juice.

#### 3.5.4 KEGG metabolic pathway analysis

**Fig. 10:**
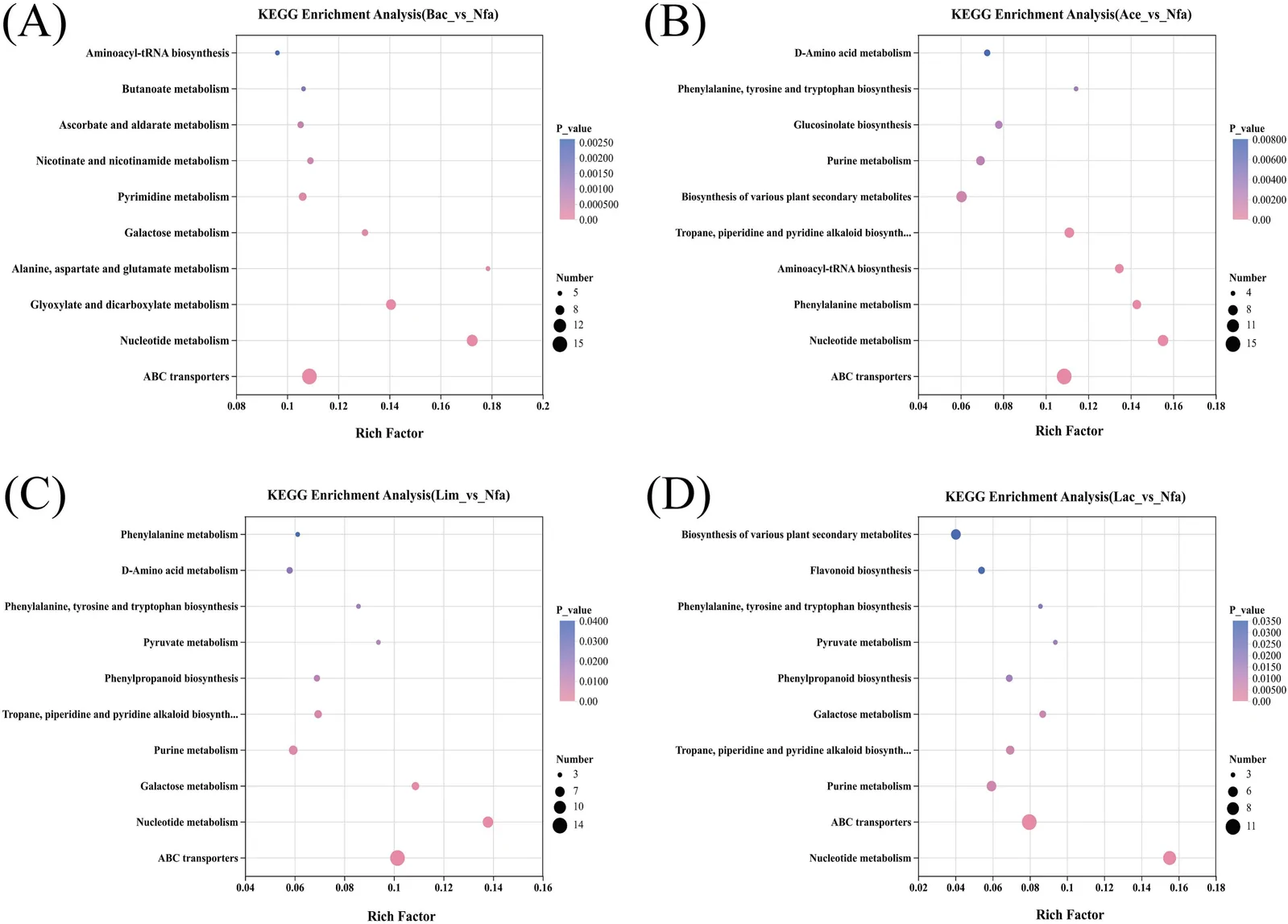
KEGG pathway enrichment bubble plot of *A. squamosa* fermentation broth.

KEGG enrichment analysis (Fig. 10A–D) revealed that all fermentation treatments significantly affected multiple metabolic pathways, particularly those related to transport and primary metabolism. In Bac vs Nfa (Fig. 10A), enriched pathways were mainly associated with central carbon and energy metabolism, including ABC transporters, nucleotide metabolism, and glyoxylate and dicarboxylate metabolism. In Ace vs Nfa (Fig. 10B), in addition to shared pathways, ABC transporters, phenylalanine metabolism, and aminoacyl-tRNA biosynthesis were significantly enriched. In Lim vs Nfa (Fig. 10C), major enriched pathways included ABC transporters, nucleotide metabolism, galactose metabolism, and purine metabolism. In Lac vs Nfa (Fig. 10D), enrichment was observed in nucleotide metabolism, ABC transporters, purine metabolism, phenylpropanoid biosynthesis, and flavonoid biosynthesis. All fermentation systems consistently targeted ABC transporters and core metabolic pathways (carbohydrate, amino acid, and nucleotide metabolism), while some strains additionally activated secondary metabolite-related pathways such as phenylpropanoid and flavonoid biosynthesis. These metabolic alterations indicate that microbial fermentation reshaped both primary and secondary metabolic processes, which may contribute to the improved nutritional composition and in vitro bioactivities of fermented *A. squamosa* juice.

#### 3.5.5 Correlation analysis between key metabolites and bioactivities

**Fig. 11:**
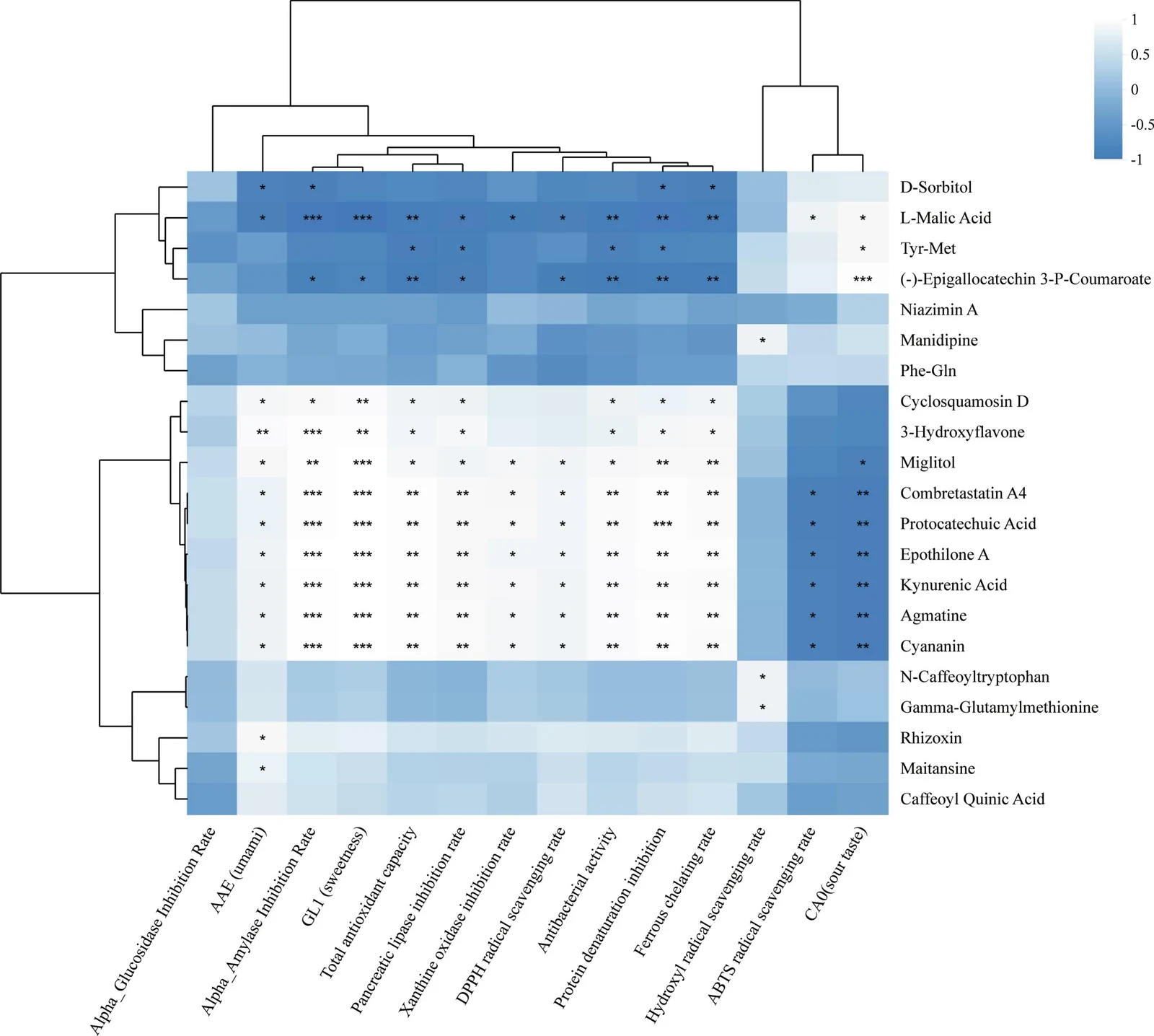
Correlation analysis between key differential metabolites and bioactivities of fermented *A. squamosa* juice.

To investigate the relationships between key metabolites (Supplementary Table S2) and the bioactivities of fermented *A. squamosa* juice, Pearson correlation analysis was performed using representative metabolites and the measured functional parameters (Fig. 11). Most phenolic compounds, amino acid derivatives, and microbial metabolites showed positive correlations with antioxidant capacity, enzyme inhibitory activities, and antimicrobial activity. Notably, protocatechuic acid, epothilone A, cyananin, kynurenic acid, agmatine, and 3-hydroxyflavone exhibited significant positive correlations with multiple antioxidant and enzyme inhibitory activities, including FRAP, DPPH radical scavenging, α-amylase inhibition, α-glucosidase inhibition, pancreatic lipase inhibition, xanthine oxidase inhibition, protein denaturation inhibition, and Fe^2+^ chelation (*P* < 0.05). Protocatechuic acid and 3-hydroxyflavone have been widely reported to possess potent antioxidant properties, whereas kynurenic acid and agmatine are associated with antioxidant and anti-inflammatory activities, supporting their potential contributions to the enhanced in vitro bioactivities observed after fermentation. In contrast, D-sorbitol, L-malic acid, Tyr-Met, and (−)-epigallocatechin 3-p-coumaroate showed negative correlations with most functional parameters but were positively associated with sourness. Overall, these findings indicate that the improved in vitro bioactivities of fermented *A. squamosa* juice may be associated with the accumulation of phenolic compounds, amino acid derivatives, and other fermentation-derived metabolites.

## 4 Discussion

Microbial fermentation has been widely recognized as an effective strategy to improve the nutritional quality, bioavailability, and sensory properties of plant-based foods. In the present study, fermentation with four functional microorganisms significantly altered the physicochemical characteristics, metabolite composition, sensory attributes, and in vitro bioactivities of *A. squamosa* juice, demonstrating clear strain-dependent differences in fermentation performance.

Prior to industrial application, microbial safety evaluation is essential. During fermentation, microorganisms may produce metabolites potentially associated with toxicity or antibiotic resistance. Therefore, hemolytic activity and antibiotic susceptibility tests are commonly used to assess biosafety. In this study, *B. subtilis*, A., *L. reuteri*, and L. *buchneri*—all widely used food-grade fermentation strains—were selected. None of the strains exhibited hemolytic activity, and all showed acceptable antibiotic sensitivity profiles. Excessive accumulation of biogenic amines is an important safety concern in fermented foods. In the present study, biogenic amine levels were not quantitatively determined because the primary objective was to investigate the effects of different functional microorganisms on the physicochemical properties, in vitro bioactivities, sensory characteristics, and metabolomic profiles of fermented *A. squamosa* juice, rather than to comprehensively evaluate all potential food safety-related metabolites. The selected microorganisms are food-associated strains, and previous studies have demonstrated that biogenic amine production is highly strain-dependent and influenced by microbial characteristics, substrate composition, and fermentation conditions (Gardini et al., 2016; Moniente et al., 2022). Some strains of *Limosilactobacillus reuteri* and *Lactobacillus buchneri* have shown limited potential for biogenic amine formation, whereas *Bacillus subtilis* and Acetobacter species used in food fermentation have been less frequently associated with substantial biogenic amine accumulation under appropriate fermentation conditions (Buňková et al., 2013; Ruiz-Capillas & Herrero, 2019). However, considering the strong strain- and substrate-dependent characteristics of biogenic amine production, targeted determination of major biogenic amines using HPLC or LC–MS/MS should be included in future studies to provide a more comprehensive safety evaluation of fermented *A. squamosa* juice.

Fermentation markedly modified the sensory characteristics of *A. squamosa* juice. The increased sourness was primarily associated with the accumulation of organic acids, whereas the enhanced umami perception was likely related to the release of free amino acids and low-molecular-weight peptides generated through microbial proteolysis. In contrast, the reductions in bitterness and astringency may be attributed to the hydrolysis of phenolic glycosides, structural transformation of phenolic compounds, and degradation of bitter substances. These compositional changes improved the overall flavor balance and may enhance consumer acceptability. Functional microbial fermentation enhances the utilization potential of *A. squamosa* juice by promoting bioactive compound accumulation and improving sensory quality, supporting its application in plant-based fermented beverages. *Bacillus subtilis* exhibits strong extracellular enzyme-producing capacity, including proteases, amylases, and polysaccharide-degrading enzymes, which may facilitate substrate degradation and contribute to enhanced functional properties(Papagianni, 2012). In contrast, Acetobacter spp. promote organic acid production, particularly acetic acid, through oxidative metabolism, thereby improving acidity regulation and flavor development(Wu et al., 2018).

The enhanced in vitro functional properties of fermented *A. squamosa* juice may be attributed to microbial-mediated cell wall degradation, release of bound phenolics, and transformation of low-molecular-weight metabolites during fermentation. Extracellular enzymes, including cellulases, pectinases, and β-glucosidases, may facilitate the liberation of polyphenols, flavonoids, and other bioactive compounds. Pearson correlation analysis further indicated that the enhanced activities were closely associated with phenolic compounds and amino acid-derived metabolites. In particular, protocatechuic acid, cyanidin derivatives, and γ-glutamylmethionine showed positive correlations with antioxidant and enzyme inhibitory activities, consistent with their reported bioactivities. Although epothilone A was positively correlated with several functional indices, its increased abundance should be interpreted as an association rather than evidence of de novo biosynthesis. The enhanced antimicrobial activity may result from the combined effects of organic acids and other microbial metabolites, while increased inhibition of α-glucosidase, α-amylase, pancreatic lipase, and xanthine oxidase further indicates improved in vitro functional potential. However, these findings are based solely on biochemical assays and require validation through targeted metabolomics and in vivo studies.

Notably, epothilone A was significantly increased in multiple fermentation groups. As a macrocyclic lactone with microtubule-stabilizing activity, epothilone A induces G2/M cell cycle arrest and exhibits antiproliferative effects(Bollag et al., 1995). Its detection in fermented *A. squamosa* juice suggests that fermentation may promote precursor transformation or alter metabolite accumulation, although further validation is required. Previous omics studies have also demonstrated that microbial fermentation can enhance phenolic bioavailability; for example, *Lactobacillus plantarum* fermentation of sea buckthorn juice increased free phenolic content by promoting glycosidase-mediated conversion of bound phenolics(Ju et al., 2025).

Overall, *B. subtilis*, *A. tropicalis*, *L. reuteri*, and L. *buchneri* induced distinct strain-specific changes in the nutritional composition, sensory characteristics, and metabolite profiles of *A. squamosa* juice, providing a basis for strain selection and process optimization. Nevertheless, enzyme inhibition assays represent simplified models of physiological functions, and further studies integrating targeted metabolite validation, bioavailability assessment, and animal experiments are needed to confirm the biological significance of these findings.

## 5 Conclusion

This study investigated the effects of fermentation with four functional microorganisms on the physicochemical properties, in vitro bioactivities, sensory characteristics, and metabolomic profiles of *A. squamosa* juice. Fermentation significantly increased total phenolic content (1.5–2.2-fold) and enhanced in vitro antioxidant, antimicrobial, and enzyme inhibitory activities. Untargeted metabolomics revealed strain-dependent alterations in metabolite profiles, with increased levels of bioactive compounds such as anthocyanin derivatives, protocatechuic acid, and γ-glutamylmethionine. Electronic tongue analysis indicated improved flavor characteristics after fermentation, mainly through enhanced sourness and umami responses and reduced bitterness and astringency. Epothilone A exhibited increased relative abundance in multiple fermentation groups; however, its metabolite identity, actual concentration, and biological significance require further confirmation through targeted quantification and functional validation. Pearson correlation analysis indicated that the enhanced in vitro activities were significantly associated with the accumulation of phenolic compounds, amino acid derivatives, and microbial metabolites. Different microorganisms exhibited strain-dependent metabolic regulation patterns, with *B. subtilis* and L. *reuteri* fermentation groups showing relatively stronger activities in certain enzyme inhibition and antimicrobial assays. Overall, functional microbial fermentation effectively modulated the nutritional composition, metabolite profile, sensory quality, and in vitro bioactivities of *A. squamosa* juice, providing experimental support for its further processing and development as a fermented functional beverage. Nevertheless, the physiological relevance of these in vitro activities and metabolite–bioactivity associations requires further validation through targeted metabolite quantification, cellular models, and animal studies.

## CRediT authorship contribution statement

**Yi Deng:** Writing – review & editing, Writing – original draft, Data curation. **Richan Fang:** Writing – original draft, Data curation. **Jun Guo:** Writing – original draft, Funding acquisition. **Chunhui Miao:** Supervision. **Yajuan Chen:** Supervision. **Xiaohuan Ke:** Project administration. **Jianmin Tang:** Project administration. **Jiating Lin:** Project administration. **Youlin Cheng:** Project administration. **Cuixia Zhao:** Supervision. **Jian Xiong:** Supervision. **Xianyu Deng:** Supervision. **Weiwei Li:** Resources, Funding acquisition. **Conghui Ji:** Writing – review & editing, Funding acquisition, Conceptualization. **Kai Qian:** Writing – review & editing, Resources, Funding acquisition, Conceptualization.

## Funding

The work was supported by Yunnan Fundamental Research Projects (grant NO. 202401AT070021); National Natural Science Foundation of China (32260863); Medical Joint Special Project of Kunming University of Science and Technology(KUST-KH2022019Y); Construction Project of Microbial Fermentation Feed Technology Innovation Team in Zunyi City(Zunyi KCTD055); Yunnan Province Expert Workstation (202405AF140081) and Yunnan International Joint Center of Urban Biodiversity (202403AP140026).

## Declaration of competing interest

The authors declare that they have no known competing financial interests or personal relationships that could have appeared to influence the work reported in this manuscript.

## Data Availability

Data will be made available on request.
