# Supplementary material for: Comparative analysis of physicochemical properties, functional activities, and metabolomics of Annona squamosa juice fermented by different functional microorganisms
Source: Food Chem X. 2026 Aug 22;39:104287. doi: 10.1016/j.fochx.2026.104287 (PMC13594634; doi:10.1016/j.fochx.2026.104287)
Supplement: Supplementary file 1 [file mmc1.docx]

**SUPPLEMENTARY INFORMATION**

**Comparative Analysis of Physicochemical Properties, Functional Activities, and Metabolomics of** ***Annona squamosa* Juice Fermented by Different Functional Microorganisms**

Table S1. Fermentation parameters of Annona squamosa juice fermented by four functional microbial strains.

| Parameter | *Bacillus subtilis* (Bac) | *Acetobacter tropicalis* (Ace) | *Limosilactobacillus reuteri* (Lim) | *Lactobacillus buchneri* (Lac) |
| --- | --- | --- | --- | --- |
| Substrate | Annona squamosa juice | | | |
| Fruit-to-water ratio (w/v) | 3:7 | | | |
| Pre-fermentation treatment | 65 ℃, 30 min (water bath), rapid cooling to room temperature | | | |
| Anti-browning agent | 0.05% (w/v) L-ascorbic acid | | | |
| Volume per flask | 100 mL in 250 mL flask | | | |
| Pre-culture medium | MRS broth | | | |
| Pre-culture conditions | 37 ℃, 24 h | | | |
| Seed liquid concentration (OD₆₀₀) | 1.0 | | | |
| Initial inoculum size (CFU/mL) | ~1.0 × 10⁷ | | | |
| Inoculation ratio (v/v) | 5% | | | |
| Fermentation temperature (℃) | 37 | | | |
| Fermentation duration (h) | 48 | | | |
| Oxygen condition | Aerobic | | | |
| Agitation speed (rpm) | 150 | | | |
| Sampling time points (h) | 0, 4, 8, 16, 24, 36, 48 | | | |
| Control group | Non-fermented Annona squamosa juice (Nfa), subjected to identical pasteurization and storage conditions | | | |
| Biological replicates | n = 3 per group | | | |

Table S2. List of key metabolites for correlation analysis in this article.

| ranking | compound name | VIP value | P-value |
| --- | --- | --- | --- |
| 1 | Epothilone A | 3.908 | 0.01177 |
| 2 | Rhizoxin | 2.619 | 0.01321 |
| 3 | Phe-Gln | 2.3856 | 0.01177 |
| 4 | Tyr-Met | 2.3152 | 0.0203 |
| 5 | Protocatechuic Acid | 2.2071 | 0.01212 |
| 6 | 3-Hydroxyflavone | 2.1749 | 0.009074 |
| 7 | Cyananin | 2.1592 | 0.009074 |
| 8 | Cyclosquamosin D | 2.0942 | 0.01177 |
| 9 | Gamma-Glutamylmethionine | 2.0401 | 0.01177 |
| 10 | (-)-Epigallocatechin 3-P-Coumaroate | 1.9878 | 0.01212 |
| 11 | D-Sorbitol | 1.9496 | 0.01019 |
| 12 | Agmatine | 1.9202 | 0.009074 |
| 13 | L-Malic Acid | 1.9178 | 0.009074 |
| 14 | Combretastatin A4 | 1.87 | 0.01212 |
| 15 | N-Caffeoyltryptophan | 1.825 | 0.0221 |
| 16 | Manidipine | 1.7922 | 0.0144 |
| 17 | Kynurenic Acid | 1.7884 | 0.01662 |
| 18 | Maitansine | 1.7882 | 0.04708 |
| 19 | Miglitol | 1.7697 | 0.02406 |
| 20 | Niazimin A | 1.7627 | 0.01863 |
| 21 | Caffeoyl Quinic Acid | 1.741 | 0.02619 |

Table S3. PLS-DA model parameters of samples.

| A | R2X | R2X(cum) | R2Y | R2Y(cum) | Q2 | Q2(cum) |
| --- | --- | --- | --- | --- | --- | --- |
| p1 | 0.412 | 0.412 | 0.25 | 0.25 | 0.161 | 0.161 |
| p2 | 0.179 | 0.591 | 0.246 | 0.496 | 0.139 | 0.278 |
| p3 | 0.112 | 0.703 | 0.25 | 0.745 | 0.238 | 0.45 |
| p4 | 0.0466 | 0.893 | 0.243 | 0.992 | 0.863 | 0.943 |

Table S4. Statistics of the number of variables.

| **Ion mode** | **All peaks** |
| --- | --- |
| pos | 12563 |
| neg | 8995 |
| mix | 21558 |

Figure S1. Chemical Composition of *A. squamosa*. (A)-(B): chromatogram analysis;

(C)-(J): mass spectrogram analysis.

Figure S2. Absorption spectra (200–750 nm) of all samples fermented for 24 and 48 hours. (A)-(E): control group, *B. subtilis* group, *A. tropicalis* group, *L. reuteri* group, *L. buchneri* group.
